# The Mechanism and Experimental Validation of Forsythoside A in the Treatment of Male Infertility Were Analyzed Based on Network Pharmacology and Molecular Docking

**DOI:** 10.1155/2022/7723358

**Published:** 2022-10-06

**Authors:** Zhen Ma, Xueling Liu, Haiwang Lu, Haoming Li, Ruizhi Gao, Rong Wen, Zhiping Tang, Haihui Yin, Yun He, Hong Yang

**Affiliations:** ^1^Department of Medical Ultrasound, The First Affiliated Hospital of Guangxi Medical University, Nanning, Guangxi Zhuang Autonomous Region, China; ^2^Department of Medical Ultrasound, Guangxi International Zhuang Medical Hospital, Nanning, Guangxi Zhuang Autonomous Region, China; ^3^Department of Medical Ultrasound, The First Affiliated Hospital of Guangxi University of Chinese Medicine, Nanning, Guangxi Zhuang Autonomous Region, China; ^4^Department of Andrology, The First Affiliated Hospital of Guangxi University of Chinese Medicine, Nanning, Guangxi Zhuang Autonomous Region, China; ^5^Guangxi University of Chinese Medicine, Nanning, Guangxi Zhuang Autonomous Region, China

## Abstract

Chinese medicine extracts are currently the hotspot of new drug research and development. Herein, we report the mechanism of action of the traditional Chinese medicine extract Forsythiaside A in the treatment of male infertility and experimental verification. We first obtained 95 intersection genes between the target protein of Forsythiaside A and the target genes of male infertility and screened 13 key genes. In molecular docking, Forsythiaside A can each have a higher total docking score with 12 key genes and have a better combination. These 95 intersection genes are mainly related to biological processes such as response to peptide hormone, response to oxidative stress, and participation in the oxidative stress of the forkhead box O (FoxO) signaling pathway. Therefore, we use ornidazole to induce an experimental model of oligoasthenospermia in rats and use different concentrations of Forsythiaside A to intervene. We proved that the semen quality and superoxide dismutase (SOD) activities of model group rats were significantly lower than those of the blank group, and semen quality and SOD activities of the low-dose group and high-dose group were significantly higher than those of the model group. The malondialdehyde (MDA) level of model group rats was significantly higher than that of blank group, while the MDA levels of the low-dose group and high-dose group were significantly lower than that of the model group. Forsythoside A is a potential drug substance for male infertility and improves the semen quality, MDA levels, and SOD activities of rats with oligoasthenospermia.

## 1. Introduction

Reproductive health is the guarantee for the continued prosperity and progress of human and social development, and infertility seriously endangers human reproductive health. According to reports, about 20% of couples in the world are infertile, and 40–60% of them are caused by men [1]. The decline in male fertility involves a variety of causes, including genetics, immune factors, mitochondrial dysfunction, endocrine disorders, gonad infections, urogenital system abnormalities, varicocele, and so on [2–8]. The main method of treating male infertility is medication, including hormones, antioxidants, carnitines, trace elements, and vitamins [9–12]. However, due to its complex pathogenic factors and unclear etiological mechanisms, the treatment of male infertility has not been able to withstand the test of evidence-based medicine, and its efficacy needs to be further improved. Therefore, research on male infertility is still one of the clinical concerns.

The research of Chinese medicine on male infertility has gone through a long time, with a solid theoretical foundation and long-term clinical practice [13]. Traditional Chinese Medicine believes that “the kidney governs the production of sperm,” so the treatment is centered on the “kidney,” but it is not simply “tonifying the kidney and producing sperm,” but also needs to start with its pathogenesis. Patients with damp heat are caused by eating spicy food, drinking more alcohol, gonadal infections, and sexual incontinence; patients with poisoning are affected by harmful substances; patients with worms are infected by pathogenic microorganisms. Therefore, for the treatment of male infertility, attention should be paid not only to the treatment of kidney deficiency but also to the treatment of the cause of the disease.

Forsythia, a traditional Chinese medicine, is a heat-clearing and detoxifying drug, which is the cause of male infertility (damp heat, blood stasis, poison, insects). In-depth research on Forsythia suspensa found that its modern pharmacology is mainly anti-inflammatory. Among them, Forsythoside A is a phenethanol glycoside compound extracted from the dried fruits of Forsythia suspensa, which has significant antibacterial, antiviral and anti-oxidant effects [14, 15]. However, there is no research report on the effect and curative effect of Forsythia or Forsythoside A on male infertility.

Network pharmacology is based on systems biology and multidirectional pharmacology. Its characteristics are holistic and systematic. It can integrate the drug action network with the biological network, and recognize new drugs from the structure and function of the biomolecular network [16]. It is a powerful tool for research on traditional Chinese medicine [17]. Molecular docking is a new method of drug design [18]. It designs drugs on the basis of receptor structure, which can be used to screen potential compounds, screen possible active ingredients, and preliminarily reveal the medicinal material basis for the effects of traditional Chinese medicine.

This topic aims to elucidate the material basis and targets of Forsythoside A in the treatment of male infertility through the use of network pharmacology research methods, combined with modern molecular biology techniques and animal experiments, from a holistic and systematic perspective. The molecular mechanism of Forsythoside A in the treatment of male infertility is preliminarily clarified. It aims to provide ideas and methods for the study of effect substances and molecular mechanisms of traditional Chinese medicine, and to provide a reference for the research and development of new drugs for male infertility.

## 2. Materials and Methods

### 2.1. Screening of Drug Targets

The chemical structure of Forsythoside A was obtained from PubChem database (https://pubchem.ncbi.nlm.nih.gov/). According to the chemical structure of Forsythoside A, the pharmacophore of Forsythoside A was obtained through the PharmMapper database (http://www.lilab-ecust.cn/pharmmapper/). Then, search the corresponding gene name in the Protein Data Bank (PDB) database (https://www.rcsb.org/) according to the PDB id of Forsythoside A.

### 2.2. Screening of Disease Targets for Male Infertility

The GeneCards database (https://www.genecards.org/) and the Online Mendelian Inheritance in Man (OMIM) database (https://omim.org/) were searched to screen gene targets related to male infertility diseases. A dataset of genetic targets for male infertility was established.

### 2.3. Venn Analysis and Biomolecular Network Regulation Model

The VennDiagram package in the *R* software was used to analyze the intersection of drug target proteins and male infertility gene targets (Venn analysis) to obtain common genes. The common genes were imported into the String database (https://string-db.org/) for protein-protein interaction network (PPI) analysis to construct an internal interaction network of common genes. The interaction network was imported into Cytoscape software to filter the degree of freedom of nodes in the network. Nodes with an average degree of freedom of more than 2 times were used as key targets.

Gene ontology (GO) annotates and classifies genes according to Biology Process (BP), Molecular Function (MF), and cellular component (CC). Kyoto Encyclopedia of Genes and Genomes (KEGG) is a database for genome deciphering. Given a complete set of genes in chromosomes, it can predict the role of protein interaction networks in various cellular activities. More advanced functional information is stored in the KEGG PATHWAY database, including graphical cell biochemical processes such as metabolism, membrane transport, signal transmission, cell cycle, and conserved sub-pathways. The clusterprofiler package of *R* software (4.0.2 version) was used for GO function analysis and KEGG pathway enrichment analysis. By analyzing the degree of enrichment of key genes, the GO term of key genes and the *p*-value of the KEGG Pathway are calculated, and the biological functions and signal pathways with significant enrichment of key genes are screened out.

### 2.4. Molecular Docking

PubChem database was used to obtain Forsythoside A related molecular structure (sdf format), which was converted to mol2 format by Open Babel GUI software. The PDB database was used to obtain the protein crystal structure (pdb format). It was imported into Sybyl X2.0 software to optimize the structure and find the active site of the target protein. The compound structure and crystal structure were simultaneously imported into Sybyl X2.0 software for molecular docking. The Total Score of the molecular docking module is greater than 6.0, indicating that the ligand has a better ability to bind to the receptor.

### 2.5. Experimental Animals

A total of 36 Sprague-Dawley (SD) male rats (sexually mature), 8-week old, weighing 170–190 g, were purchased from Hunan Changsha Tianqin Biotechnology Co., Ltd. They were kept in the Clinical Experiment Center of Traditional Chinese Medicine of Guangxi University of Traditional Chinese Medicine and kept by special breeders for 1 week. Rats can freely ingest solid feed and deionized water, and the temperature and humidity are constant. There were no obvious abnormalities in 36 rats. The experimental protocol on animals in this experiment was approved by the Experimental Animal Ethics Committee of the First Affiliated Hospital of Guangxi Medical University (Ethics approval number: NO. 2022-KY-E-(285)).

### 2.6. Main Experimental Reagents

Forsythoside A (purity: >98%; specification: 20 mg; purchased from Chengdu Refines Biotechnology Co., Ltd.; CAS: 79916-77-1). Ornidazole (batch number: B200601; dosage form: tablet; specification: 0.25 g × 24 tablets; purchased from Huadong Medicine Bohua Pharmaceutical Co., Ltd.).

### 2.7. Establishment of the Rat Infertility Model and Grouping

After the rats were raised for one week, 36 rats were numbered, weighed (electronic scale), and recorded. Then the rats were randomly (Random number table) divided into four groups (nine in each group), namely the blank group, model group, low dose group, and high dose group. After that, in addition to regular feeding, each group took the following measures: Blank group: gavage treatment with normal saline. Model group: gavage treatment with 400 mg/(kg·day) ornidazole. Low dose group: gavage treatment with 400 mg/(kg·d) Ornidazole + 10 mg/kg Forsythoside A. High dose group: gavage treatment with 400 mg/(kg·d) Ornidazole + 20 mg/kg Forsythoside A.

All rats were given medicine at the same time every day, and the medicine intervention time was 3 weeks. The rats were weighed once a week, and the dose of the drug was adjusted according to changes in body weight. During the experiment, the rats ingest food freely, and the ambient temperature was kept at 20–25°C, and the relative humidity was 50%–65%. During the experiment, the rats' body weight, diet, urination and defecation, body hair, and changes in the mental state should be closely observed.

### 2.8. Detection of Rat Semen Quality

Twenty-four hours after the last administration, all rats were weighed and recorded. All rats were collected at the same time of day. The rats were anesthetized by intraperitoneal injection of ketamine and then killed by cervical dislocation. The abdominal cavity of the rat was quickly opened with tissue scissors, and the testis and epididymis tissues were quickly peeled off. Semen quality analysis: rat epididymal tissues (all left tissues) were placed in 2 mL of normal saline. The epididymal tissue was cut into pieces with tissue scissors, and then placed in a 37°C constant temperature water bath to mix sperm and physiological saline. The glass slide was dropped with 1 drop (0.5 ml) of sperm mixture and placed in a computer-aided sperm analyzer (Hamilton Thorne, United States Of America). Choose 5 fields of view to calculate sperm concentration (10^6^ mL), the percentage of forward movement of sperm (PR, %), total sperm motility (PR + non-forward movement of sperm (NP), %), sperm deformity rate (%), and calculate the average value.

### 2.9. Detecting the Level of Lipid Peroxide Malondialdehyde (MDA) and the Activity of Superoxide Dismutase (SOD) in Rats

The epididymal tissue on the right was removed, and the epididymal tissue was added to 0.9% NaCl solution to make a 5% tissue homogenate in an ice water bath at 4°C. Put the homogenate in a centrifuge and centrifuge at 3000 r/min for 10 min. The supernatant was used to detect the MDA content and SOD activity, and the MDA content and SOD activity were determined according to the operating method of the kit instructions. Both MDA content kit and SOD kit were purchased from Suzhou Keming Biotechnology Co., Ltd. SOD activity (xanthine oxidase method): According to the procedure of the kit instructions, mix the reagents thoroughly according to the prescribed dosage and let them stand for 5 minutes, then 1 ml was added to a glass cuvette and measure the absorbance of each tube at 560 nm. SOD activity (U/mg) = inhibition percentage/(1-inhibition percentage)/sample protein concentration (mg/ml), where inhibition percentage = (control tube absorbance - measuring tube absorbance)/control tube absorbance × 100%. MDA detection (thiobarbital method): Preparation of MDA detection working solution: Reagent one was added to each bottle of reagent two, dissolve and mix well, and store at 4°C until use. Accurately pipette 0.6 mL detection working solution into a 1 mL centrifuge tube, and then add 0.2 mL crude enzyme solution. After the mixture was kept in a water bath at 100°C, it was immediately cooled in an ice bath and centrifuged at 10,000 g/min for 10 minutes. Take the supernatant into a glass cuvette, and measure the absorbance of each sample at 532 nm and 600 nm. MDA content (nmol/ml) = measurement absorbance - measurement blank tube absorbance/standard tube absorbance-standard blank tube absorbance × standard product concentration (10 nmol/ml)/protein content (mgprot/ml).

### 2.10. Statistical Methods

Statistical analysis was performed with GraphPad Prism 8 software. Data (quantitative data) are expressed as mean ± standard deviation. The comparison between the two groups conforms to the normal distribution with the *t*-test, and the non-conformity analysis uses the rank sum test. One-way analysis of variance was used among multiple groups. Post-hoc comparison using the Bonferroni method. *P* < 0.05 was regarded as significant difference.

## 3. Results

### 3.1. Target Protein for Forsythoside A and Biomarker for Male Infertility

**Figure 1:**
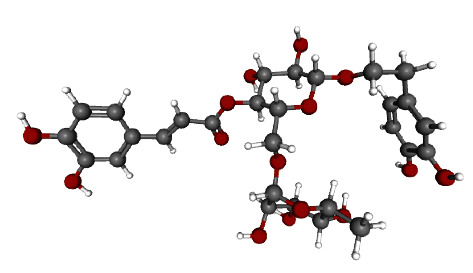
The 3D structure of forsythoside A.

The 3D structure of Forsythoside A is shown in Figure 1. Based on the PDB database, 439 target proteins corresponding to Forsythoside A were screened. A search of the GeneCards database found 4205 genes for male infertility biomarkers.

### 3.2. Common Genes of Drugs and Diseases and Their Biomolecular Network Regulation Model

**Figure 2:**
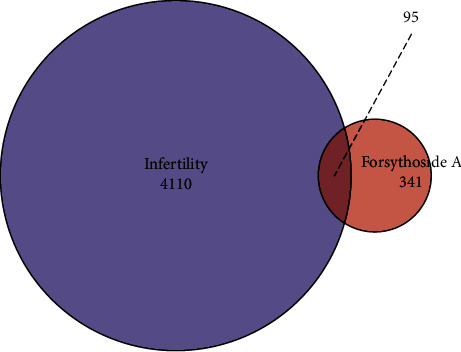
Venn analysis, 439 forsythoside A target proteins, 4205 male infertility biomarkers, and 95 common genes.

**Figure 3:**
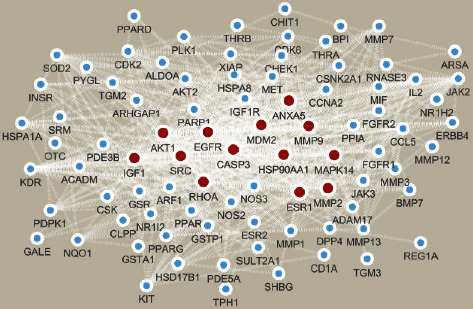
Protein interaction network diagram (red dots are key genes).

The intersection of 439 Forsythoside A target proteins with 4205 male infertility biomarkers (Venn analysis); 95 common genes were obtained (see Figure 2). 95 common genes were put into the String database to search, and the interaction information between the proteins was obtained. After inputting its interaction information into Cytoscape software, a PPI network diagram was obtained (see Figure 3), and 13 key genes were screened out (serine/threonine kinase 1 (AKT1), estrogen receptor 1 (ESR1), SRC Proto-Oncogene, Non-Receptor Tyrosine Kinase (SRC), caspase 3 (CASP3), epidermal growth factor receptor (EGFR), insulin-like growth factor 1 (IGF1), matrix metallopeptidase 9 (MMP9), heat shock protein 90 alpha family class A member 1 (HSP90AA1), ras homolog family member A (RHOA), annexin A5 (ANXA5), proto-oncogene (MDM2), mitogen-activated protein kinase 14 (MAPK14), and matrix metallopeptidase 2 (MMP2)).

### 3.3. Gene Function Enrichment Analysis

**Figure 4:**
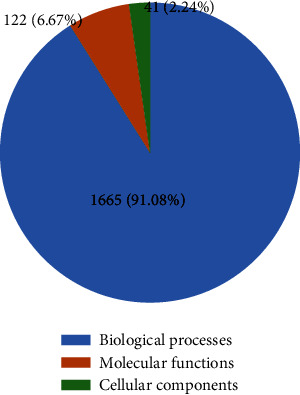
The proportion of biological processes, molecular functions, and cellular components in GO functional analysis.

**Figure 5:**
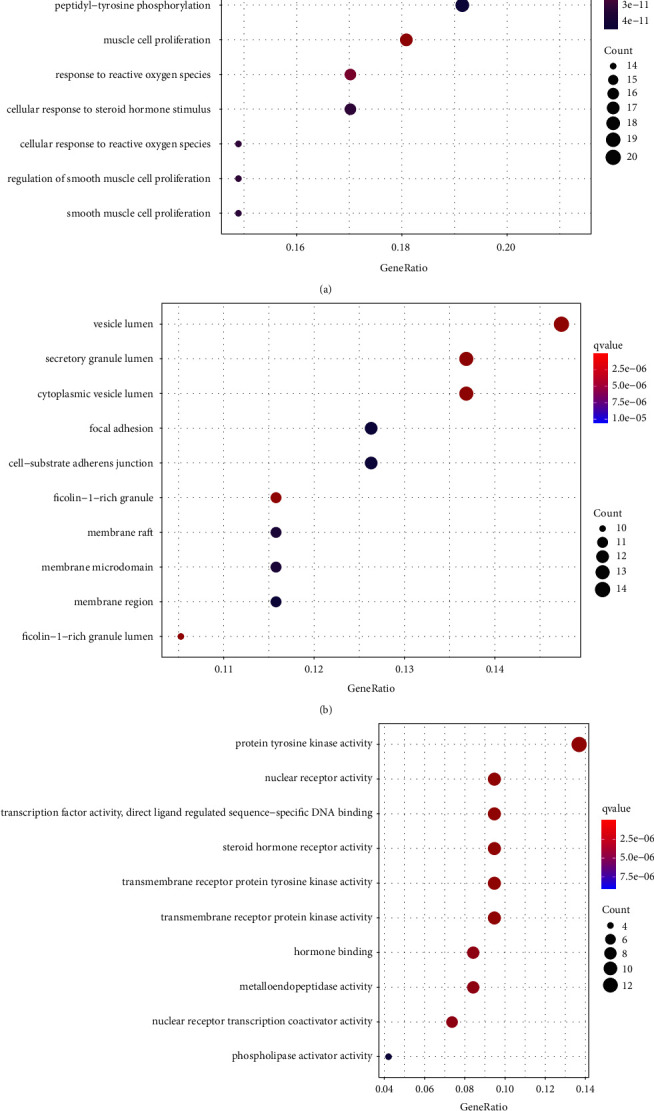
GO function analysis results: (a) biological process, (b) molecular function, and (c) cell component.

**Figure 6:**
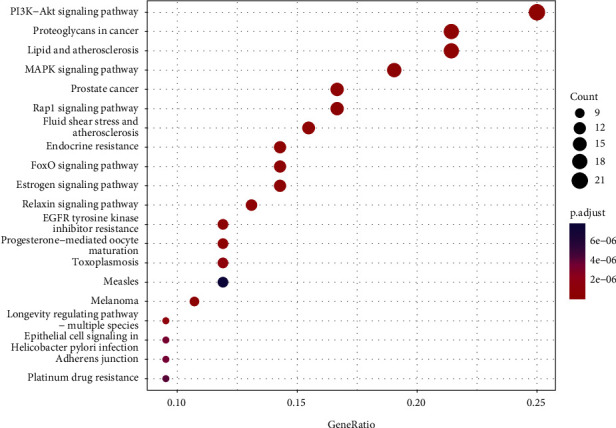
KEGG pathway enrichment analysis results.

The 95 common genes were analyzed for GO function annotation and KEGG enrichment. The GO analysis results are shown in Figure 4, in which 1665 biological processes, 122 molecular functions, and 41 cellular components are annotated. The proportion of each part is shown in Figure 5. The results show that Response to oxidative stress, Response to peptide hormone, Cellular response to oxidative stress, Peptidyl-tyrosine phosphorylation, Peptidyl-tyrosine modification, Response to steroid hormone, Cellular response to peptide, Gland development, Muscle cell proliferation, Cellular response to peptide hormone stimulus, etc., are the main biological processes involved in drug targets. Vesicle lumen, Secretory granule lumen, Cytoplasmic vesicle lumen, Focal adhesion, Cell-substrate adherens junction, Cell-substrate junction, Ficolin-1-rich granule, Membrane raft, Membrane microdomain, Membrane region, etc., are the main Cellular Component involved in drug targets. Protein tyrosine kinase activity, Endopeptidase activity, Protein serine/threonine kinase activity, Nuclear receptor activity, Transcription factor activity, Direct ligand regulated sequence-specific DNA binding, Steroid hormone receptor activity, Transmembrane receptor protein tyrosine kinase activity, Transmembrane receptor protein kinase activity, Phosphatase binding, Cell adhesion molecule binding, etc., are the main Molecular Function in drug targets. KEGG enriches 105 signal pathways, among them, phosphatidylinositol 3-kinase - serine/threonine kinase (PI3K-Akt) signaling pathway, MAPK signaling pathway, DNA-binding transcription factor (Rap1) signaling pathway, Endocrine resistance, FoxO signaling pathway, Estrogen signaling pathway, Ras signaling pathway, Relaxin signaling pathway, Focal adhesion, etc. are the main signaling pathways that Forsythoside A acts on male infertility, as shown in Figure 6.

### 3.4. Molecular Docking

**Figure 7:**
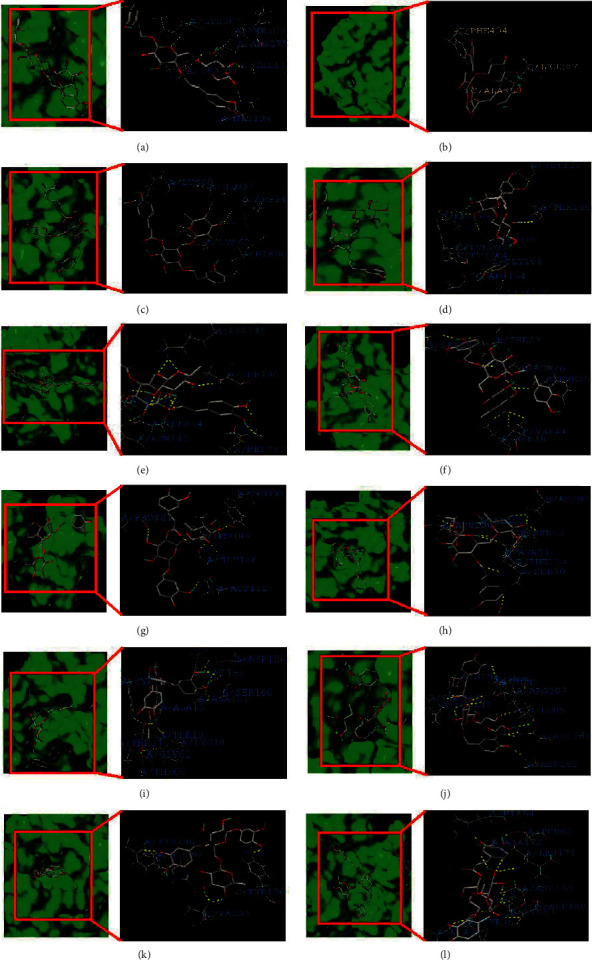
Molecular docking results. (a) AKT1, (b) ESR1, (c) SRC, (d) CASP3, (e) EGFR, (f) IGF1, (g) MMP9, (h) HSP90AA1, (i) RHOA, (j) ANXA5, (k) MDM2, and (l) MAPK14.

In order to further study the relationship between these 13 key targets (AKT1, ESR1, SRC, CASP3, EGFR, IGF1, MMP9, HSP90AA1, RHOA, ANXA5, MDM2, MAPK14, MMP2) and Forsythoside A. We used Sybyl X2.0 software for molecular docking. The crystal structures obtained from the PDB database are AKT1 (3CQU), ESR1 (1QKU), SRC (1O4I), CASP3 (3DEJ), EGFR (2ITX), IGF1 (1GZR), MMP9 (1GKD), HSP90AA1 (3EKO), RHOA (1KMQ), ANXA5 (1HAK), MDM2 (1RV1), MAPK14 (1W7H), MMP2 (1HOV). The detailed scores between receptors and ligands are shown in Table 1. Forsythoside A has higher scores with AKT1, ESR1, SRC, CASP3, EGFR, IGF1, MMP9, HSP90AA1, RHOA, ANXA5, MDM2, and MAPK14 respectively, and has a good combination (see Figures 7(a)to 7(l)).

### 3.5. Animal Experiment Verification

In order to verify the effect of Forsythiaside A on male infertility, we conducted a rat experiment. Thirty-six rats were randomly divided into 4 groups, one of which was used as a blank control; the other three groups were induced to be infertility models, and then they were intervened with different doses of Forsythiaside A. During the experiment, one SD male rat died in the Model group. It is preliminary guessed that the death of the rat may be caused by improper gavage, and has nothing to do with the drug itself. After the experiment was completed, the number of rats in each group of the Blank group, Model group, Low dose group, and High dose group were 9, 8, 9, and 9.

### 3.6. Effects of Forsythiaside A on Sperm Quality in Oligoasthenospermia Rats

**Figure 8:**
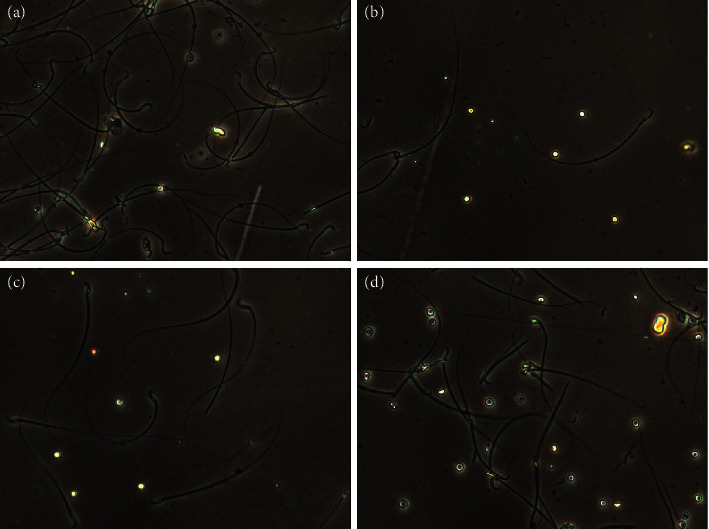
The sperm of each group was observed under the computer-assisted sperm analyzer: (a) Blank group; (b) Model group; (c) Low dose group; (d) High dose group.

Figure 8 shows the sperm of rats in each group under the computer-assisted sperm analyzer. As shown in Table 2, compared with Blank group rats, the sperm concentration, total sperm motility, and the percentage of forward sperm movement of Model group rats decreased significantly, and the rate of sperm deformity increased significantly (*P* < 0.05). Compared with Model group rats, the sperm concentration, total sperm motility, and the percentage of forward sperm movement of Low dose group and High dose group rats increased, and the rate of sperm deformity decreased (*P* < 0.05).

### 3.7. Effects of Forsythiaside A on MDA and SOD in Oligoasthenospermia Rats

**Figure 9:**
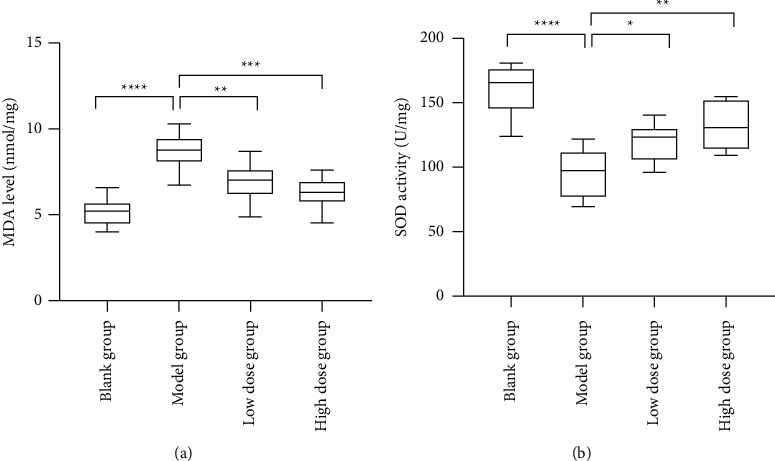
Comparison of MDA levels and SOD activities of rats in different groups. (A) MDA content; (B) SOD activity. ^*∗*^*p* < 0.05, ^*∗∗*^*p* < 0.01, ^*∗∗∗*^*p* < 0.001, and ^*∗∗∗∗*^*p* < 0.0001.

As shown in Table 3, the MDA level of Model group rats was significantly higher than that of the Blank group (*P* < 0.05). The MDA levels of Low dose group and High dose group rats were significantly lower than those of the Model group (*P* < 0.05); the specific results are shown in Figure 9(a). The SOD activity of Model group rats was significantly lower than that of the Blank group (*P* < 0.05). The SOD activity of low-dose group and high-dose group rats was significantly higher than that of the Model group (*P* < 0.05); the specific results are shown in Figure 9(b).

## 4. Discussion

Because of its complicated etiology and unclear pathogenesis, many treatment methods for male infertility have not achieved good results. Male infertility is considered to be a kidney deficiency in traditional Chinese medicine. The factors of kidney deficiency include damp heat, blood stasis, poison, and insects. Forsythia, a traditional Chinese medicine, is a heat-clearing and detoxifying medicine, which is symptomatic of male infertility. Forsythoside A is extracted from Forsythia suspensa. It is one of the strongest anti-inflammatory and anti-oxidant components in the extract of Forsythoside. At present, there are few research reports on the effect and efficacy of Forsythia or Forsythoside A on male infertility. Therefore, this study is based on network pharmacology and molecular docking analysis of the mechanism of Forsythoside A in the treatment of male infertility and experimental verification.

Through network pharmacology, this study found that Forsythoside A has 95 common genes in male infertility, including AKT1, ESR1, SRC, CASP3, EGFR, IGF1, MMP9, HSP90AA1, RHOA, ANXA5, MDM2, MAPK14, MMP2. Among them, AKT1 is the core factor of the PI3K/AKT signaling pathway. The activated AKT can be relocated to the nucleus, cytoplasm and other cell sites, phosphorylating a large number of substrate proteins. It regulates cell growth, proliferation, apoptosis, and glucose metabolism, including in germ cells. Studies have pointed out that phosphorylation of AKT can also phosphorylate mechanistic target of rapamycin kinase (mTOR), thereby regulating the formation of the blood-testis barrier and other biological processes. It also affects the biological behavior of spermatogonia and sperm formation by regulating the expression of its downstream kinases and proteins [19, 20]. In addition, the occurrence of sperm is highly dependent on the production of glucose metabolism, and the PI3K/AKT signaling pathway plays a central role in processes such as glucose metabolism [21]. If the PI3K/AKT pathway in human tissues is damaged, it can lead to insulin resistance, resulting in abnormal glucose metabolism and production capacity, which affects spermatogenesis. ESR1 (estrogen receptor 1) plays an important role in normal male reproductive development (spermatogenesis, maturation, etc.) and maintaining homeostasis [22]. Some experiments have shown that knocking out the ESR1 gene can change the efferent tubules of the mouse testis and the microenvironment of the epididymis, which in turn causes the concentration of epididymal sperm to decrease and the vitality of the epididymis [23]. SRC protein is a kind of Src family kinase, which is involved in the processes of cell metabolism, growth and development, and differentiation. SRC protein plays an important role in the development of the epididymis and the transport of sperm to the epididymal tail [24], and it also acts on sperm capacitation and acrosome reaction [25–28]. CASP3 (Caspase 3) is a key effector for the execution of apoptosis, including the apoptosis of mammalian spermatogenic cells. When a large amount of CASP3 is activated, it can cause programmed death of germ cells, which can directly reduce sperm production and reduce its quality [29]. VEGF (vascular endothelial growth factor) is a factor that promotes angiogenesis, and its testicular microcirculation has a significant impact on male reproduction. In summary, Forsythoside A may act on male infertility through the above target genes.

In this study, through GO enrichment analysis, 1665 biological processes, 122 molecular functions, and 41 cellular components were obtained. It can be seen that Forsythoside A can act on male infertility through a variety of biological functions, mainly through: Response to oxidative stress, Response to peptide hormone, Cellular response to oxidative stress, Peptidyl-tyrosine phosphorylation, Peptidyl-tyrosine modification, Response to steroid hormone, Cellular response to peptide, Gland development, Muscle cell proliferation, Cellular response to peptide hormone stimulus, and other biological processes. Vesicle lumen, Secretory granule lumen, Cytoplasmic vesicle lumen, Focal adhesion, Cell-substrate adherens junction, Cell-substrate junction, Ficolin-1-rich granule, Membrane raft, Membrane microdomain, Membrane region, and other Cellular Component. Protein tyrosine kinase activity, Endopeptidase activity, Protein serine/threonine kinase activity, Nuclear receptor activity, Transcription factor activity, Direct ligand regulated sequence-specific DNA binding, Steroid hormone receptor activity, Transmembrane receptor protein tyrosine kinase activity, Transmembrane receptor protein kinase activity, Phosphatase binding, Cell adhesion molecule binding, and other Molecular Function. KEGG enrichment analysis found that PI3K-Akt signaling pathway, MAPK signaling pathway, Rap1 signaling pathway, Endocrine resistance, FoxO signaling pathway, Estrogen signaling pathway, Ras signaling pathway, Relaxin signaling pathway, Focal adhesion, and other signaling pathways are the main signaling pathways of forsythin A in male infertility.

With the in-depth study of the PI3K-Akt signaling pathway, it has been found that activation of this pathway is closely related to the prognosis of spermatogenesis, maturation, and even male infertility. Research by Feng et al. showed that PI3K-Akt signal activation accelerates the process of G1 and S phases, thereby accelerating the cell cycle [30]. Studies have shown that PI3K has a negative regulatory effect on human sperm motility, and its large expression can reduce sperm motility and motility [31]. PI3K inhibitors can significantly improve sperm motility and exercise capacity in patients with asthenospermia [32]. The Mitogen-activated Protein Kinas (MAPK) signaling pathway can participate in a variety of cellular activities through signal transduction. The MAPK family includes ERK (Extracellular Regulated Protein Kinases), JNK (c-JunN-terminal Kinase/stress-activated Protein Kinase) and p38. The activation of ERK plays a key role in spermatogenesis and its functions (including spermatogonia division, meiosis, sperm capacitation). Almog et al. [33] showed that ERK1/2 and P38 can regulate sperm motility. In addition, multiple studies have found that excessive activation of JNK, ERK, and p38 can affect the integrity of the blood-testis barrier [34–37]. Rap1 in the Rap1 signaling pathway can recruit many active proteins after extracellular stimulation, thereby regulating cell growth, apoptosis, secretion and adhesion and other processes. Relevant animal experiments have shown that Rap1 is located differently after the meiosis of spermatogenic cells, and has a great influence on the hormone-transmission-related pathways of the spermatogenesis process, which may mainly act through the regulation of hormones [38]. And some experiments have shown [39] that Rap1 mutations can cause spermatogenesis barriers, that is, blocking the Rap1 pathway leads to support cell adhesion barriers and premature release, resulting in animal infertility. And clinical studies have found that the Rap1 mRNA in the spermatogenic cells of male infertility is abnormally high [40, 41], which inhibits sperm production. The FOXO signaling pathway is involved in oxidative stress to cells and cell cycle apoptosis. FOXO1 in the FOXO family is also a direct signaling molecule downstream of the PI3K/Akt pathway. When PI3K/AKT is activated, it can promote FOXO1 phosphorylation and bind to other proteins, translocate out of the nucleus, resulting in the loss of FOXO1 transcriptional activity, promote cell proliferation, and reduce apoptosis [42]. Research by Shen et al. [43] also showed that FOXO1-related signaling pathways inhibit cell apoptosis. This is conducive to the anti-apoptotic effect of abnormal apoptosis of germ cells. Estrogen in the Estrogen signaling pathway can induce sperm activation and regulate sperm production by regulating germ cell apoptosis [44]. Ras signaling pathway is an important cell conduction signaling pathway, which can regulate a variety of cell physiological functions, including spermatogenesis. The relaxin in the Relaxin signaling pathway is a short-circulation peptide hormone. Pimenta et al. [45] experiments showed that the effect of relaxin on the spermatogenesis process can be carried out by regulating the self-renewal of spermatogonia and promoting cell contact. This shows that Forsythoside A can act on male infertility through a variety of pathways.

In order to further explore the role of Forsythoside A in the treatment of male infertility, this study also molecularly docked Forsythoside A with 13 key targets. The results showed that Forsythoside A had a good combination with AKT1, ESR1, SRC, CASP3, EGFR, IGF1, MMP9, HSP90AA1, RHOA, ANXA5, MDM2, and MAPK14, respectively. The more receptors a ligand binds, the more likely it is to be considered as a potential active ingredient. Therefore, we believe that Forsythoside A has a greater possibility as a potential drug substance for male infertility.

Based on the results of network pharmacology predictions, this study also conducted animal experiments to verify the therapeutic effect of Forsythoside A on male infertility. Oligoasthenospermia is a common type of male infertility. In this study, ornidazole was used to induce an experimental model of oligoasthenospermia in rats. After treatment with Forsythoside A, the semen quality and hormone levels of rats were observed and analyzed. The experimental results show that the sperm concentration, total sperm motility, and the percentage of forward sperm movement of Model group rats are significantly lower than those of the Blank group, and the rate of sperm deformity is significantly increased. The sperm concentration, total sperm motility, and the percentage of forward sperm movement in Low dose group and High dose group rats were higher than those of Model group.

Because Forsythoside A has anti-oxidant capacity [15], coupled with GO analysis shows that Forsythoside A can act on male infertility through biological functions such as response to oxidative stress, and KEGG analysis shows that Forsythoside A can participate in cell activation through the FOXO signaling pathway. Oxidative stress. Therefore, this study analyzed the effect of Forsythoside A on MDA level and SOD activity in rats. Relevant studies have shown that the low anti-oxidant capacity and high concentration of unsaturated fatty acids in germ cells make them susceptible to the destruction of the oxidation-antioxidant balance and the damage of free radicals and their oxidation products [46]. In this experiment, ornidazole can cause lipid peroxidation in the epididymis of rats, that is, the level of MDA is significantly higher than that of normal rats. Moreover, ornidazole can also cause the destruction of the rat epididymal self-oxidation-antioxidation balance mechanism, that is, the activity of SOD is significantly reduced, the generation of free radicals in the epididymis is increased, the ability of resisting free radicals is decreased, and the sperm function is impaired. After low-dose and high-dose Forsythoside A treatment, the level of MDA in rats was significantly reduced, and the activity of SOD was significantly increased. It is suggested that Forsythoside A can significantly increase the SOD activity in the rat epididymis, improve the anti-oxidant capacity in the rat, reduce oxidative stress damage, reduce the production of MDA, and significantly improve sperm motility.

## 5. Conclusion

Forsythoside A can act on male infertility through multiple targets and multiple signaling pathways. It has a good combination of multiple key genes and is a potential medicinal substance for male infertility. And it has been verified by animal experiments that Forsythoside A can improve the semen quality, MDA level and SOD activity of rats with oligoasthenospermia induced by ornidazole. The shortcoming of this study is that due to the limitation of experimental time, it was not possible to verify the effect of Forsythoside A on male infertility-related pathway proteins and target proteins. In the future, the mechanism of Forsythoside A on male infertility can be further studied to provide basic research basis for the treatment of male infertility.

## Figures and Tables

**Table 1:** Molecule docking score.

Gene	Total_Score	Crash	Polar	Cscore
AKT1	7.0305	−2.827	8.6786	5
ESR1	−17.8991	−33.7067	1.146	3
SRC	7.3125	−2.098	6.6662	4
CASP3	9.3616	−2.065	7.4847	1
EGFR	9.2876	−3.3863	6.8372	5
IGF1	6.7832	−2.3223	6.0236	3
MMP9	8.243	−1.4338	6.8245	4
HSP90AA1	9.263	−4.3449	5.7042	3
RHOA	7.6087	−9.4093	6.6994	4
ANXA5	9.2866	−2.7432	8.0782	2
MDM2	7.5969	−1.9179	3.7402	3
MAPK14	7.9744	−2.3765	8.5348	3
MMP2	5.0687	−5.1096	4.0368	4

**Table 2:** Comparison of semen quality of rats in different groups.

Group	n	Sperm concentration (10^6^ mL)	Total sperm motility (PR + NP, %)	Percentage of sperm forward movement (PR, %)	Sperm deformity rate (%)
Blank group	9	37.81 ± 5.03^a^	62.33 ± 5.61^a^	48.92 ± 8.26^a^	2.59 ± 1.37^a^
Model group	8	20.65 ± 4.72	26.09 ± 3.91	19.25 ± 2.98	15.62 ± 3.03
Low dose group	9	28.55 ± 3.17^a^	42.54 ± 3.65^a^	28.73 ± 3.61^a^	8.01 ± 1.25^a^
High dose group	9	35.72 ± 6.39^a,b^	45.17 ± 4.71^a,b^	33.65 ± 3.19^a,b^	3.29 ± 1.57^a,b^
F		20.640	90.200	51.580	83.580
*P*		<0.001	<0.001	<0.001	<0.001

*Note*. ^a^*P* < 0.001 means the group is compared with model group; ^b^*P* < 0.05 means the group is compared with the low dose group.

**Table 3:** Comparison of MDA levels and SOD activities in different groups of rats.

Group	n	MDA level (nmol/mg)	SOD activity (U/mg)
Blank group	9	5.19 ± 0.80^a^	161.07 ± 20.96^a^
Model group	8	8.68 ± 1.10	95.14 ± 19.97
Low dose group	9	6.90 ± 1.10^a^	118.97 ± 14.99^a^
High dose group	9	6.27 ± 0.89^a^	132.18 ± 18.17^a^
F		18.600	18.620
*P*		<0.001	<0.001

Note. ^a^*P* < 0.05 means that the group is compared with Model group.

## Data Availability

The datasets used and analyzed during the current study are available from the corresponding author on reasonable request.
